# Protein molecular structure, degradation and availability of canola, rapeseed and soybean meals in dairy cattle diets

**DOI:** 10.5713/ajas.18.0829

**Published:** 2019-02-07

**Authors:** Yujia Tian, Xuewei Zhang, Rongcai Huang, Peiqiang Yu

**Affiliations:** 1 Department of Animal Science, College of Animal Science and Veterinary Medicine, Tianjin Agricultural University, Tianjin 300384, China; 2 State Key Laboratory of Animal Nutrition, Institute of Animal Science, Chinese Academy of Agricultural Sciences, Beijing 100193, China; 3 Department of Animal and Poultry Science, College of Agriculture and Bioresources, University of Saskatchewan, 51 Campus Drive, Saskatoon, SK, S7N 5A8, Canada

**Keywords:** Chemical Profile, Molecular Spectral Feature, Nutrient Variation and Availability, Soybean Meal, Canola Meal, Rapeseed Meal

## Abstract

**Objective:**

The aims of this study were to reveal the magnitude of the differences in protein structures at a cellular level as well as protein utilization and availability among soybean meal (SBM), canola meal (CM), and rapeseed meal (RSM) as feedstocks in China.

**Methods:**

Experiments were designed to compare the three different types of feedstocks in terms of: i) protein chemical profiles; ii) protein fractions partitioned according to Cornell Net Carbohydrate and Protein System; iii) protein molecular structures and protein second structures; iv) special protein compounds-amino acid (AA); v) total digestible protein and energy values; vi) *in situ* rumen protein degradability and intestinal digestibility. The protein second structures were measured using FT/IR molecular spectroscopy technique. A summary chemical approach in National Research Council (NRC) model was applied to analyze truly digestible protein.

**Results:**

The results showed significant differences in both protein nutritional profiles and protein structure parameters in terms of α-helix, β-sheet spectral intensity and their ratio, and amide I, amide II spectral intensity and their ratio among SBM, CM, and RSM. SBM had higher crude protein (CP) and AA content than CM and RSM. For dry matter (DM), SBM, and CM had a higher DM content compared with RSM (p<0.05), whereas no statistical significance was found between SBM and CM (p = 0.28). Effective degradability of CP and DM did not demonstrate significant differences among the three groups (p>0.05). Intestinal digestibility of rumen undegradable protein measured by three-step *in vitro* method showed that there was significant difference (p = 0.05) among SBM, CM, and RSM, which SBM was the highest and RSM was the lowest with CM in between. NRC modeling results showed that digestible CP content in SBM was significantly higher than that of CM and RSM (p<0.05).

**Conclusion:**

This study suggested that SBM and CM contained similar protein value and availability for dairy cattle, while RSM had the lowest protein quality and utilization.

## INTRODUCTION

Soybean meal (SBM) is a common feedstuff in the diet of ruminants. Rapeseed can be processed into a commercial by-product called rapeseed meal (RSM), which is utilized as a protein source in ruminant diets. It is believed that the presence of glucosinolate and erucic acid in rapeseed restricts its use in ruminants [1,2]. However, others report that by prepress-solvent extraction with hexane, these compounds can be reduced in canola meal (CM) [3]. Hitherto, SBM, RSM, and CM have been utilized as protein resources in Chinese dairy industry.

Many previous research projects have focused on the chemical components and their differences among SBM, RSM, and CM or on the their effects in ruminant or monogastric animal production performance [4,5]. However, detailed analysis of the differences of SBM, RSM, and CM based on protein secondary structures is rare. Yu [6] proposed to use synchrotron technique for the analysis of inherent structure of biological tissues. This novel approach suggested that by analyzing a scanned picture of changes of protein structures in the intrinsic protein structures in terms of protein alpha-helix to beta-sheet intensity and their ratio, we can disclose the effects of different feedstocks on protein value and nutrient availability.

Our researchers have conducted several studies on feed stocks regarding protein chemical and nutrient profiles, protein utilization and availability, and protein molecular structures during recent years. Samadi and Yu [7] revealed the protein secondary structure of dry and moist soybeans. They have confirmed the spectra region (ca. 4,000 to 800 cm^−1^), fingerprint region (ca. 1,800 to 800 cm^−1^) and protein structure baseline regions (ca. 1,715 to 1,480 cm^−1^) for soybean seed. Theodoridou and Yu [8] studied protein molecular structures of canola as meal or presscake. They revealed that different processing methods resulted in variation of protein secondary structure in canola. However, there is little research which systematically analyzes the differences between RSM, SBM, and CM based on protein secondary structures in relation to protein digestive behaviors and nutritive value in the rumen and intestine in dairy cattle.

The objectives of this study were: i) to reveal the protein chemical profiles of SBM, RSM, and CM; ii) to measure the protein subfractions partitioned by Cornell Net Carbohydrate and Protein System (CNCPS); iii) to analyze special protein compounds-amino acid (AA); iv) to determine the protein molecular structures in terms of α-helix to β-sheet intensity and their ratio, amide I and amide II intensity and their ratio; v) to analyze the truly digestible protein and energy value with a summary chemical approach in NRC model; vi) to investigate the protein degradation and protein intestinal digestion using *in situ* method and three-step *in vitro* method. The hypothesis of this study was that there were significant differences in molecular structure, physiochemical and nutrient profiles among SBM, RSM, and CM, which could result in different nutritive value in ruminants.

## MATERIALS AND METHODS

### Feed source and processing

In this study, 3 SBM samples were collected from TianJin, HeiLongJiang, and HeBei province in China; 2 CM samples came from LiaoNing and FuJian province; 3 RSM samples were gathered from SiChuan, AnHui, and JiangSu province. Each of the dried samples was ground to pass a Wiley mill (0.45 mm screen) and kept in airtight plastic bags for later use in chemical analysis experiments. Some samples were ground to pass a Wiley mill (0.25 mm screen) for molecular spectroscopy tests, while other samples of feed were dried to pass through a 2.5 mm sieve for *in situ* trial.

### Animals and diets

Five Holstein heifers with an average body weight of 600±15 kg were surgically cannulated with permanent rumen fistula and served as experimental animals. All animal experiments in this study were approved by the Ethics Committee on animals of Tianjin Agricultural University (TJAUA-2016-001). Animal care and use were conducted in accordance with the practices outlined in the Guide for the Care and Use of Agriculture Animals in Agriculture Research and Teaching [9]. The heifers were fed twice daily at 06:00 and 16:00 h with a total mixed ration formulated at a 40:60 (dry matter [DM] basis) concentrate to forage ratio to satisfy 1.3 times the maintenance nutrient requirement for heifers according to NRC requirement (NRC [10]). Water was available *ad libitum*.

### Rumen degradation procedure

To compare the degradability among SBM, CM, and RSM, *in situ* ruminal DM and crude protein (CP) degradability were determined following the method of Ørskov and McDonald [11] and Theodoridou and Yu [12]. Seven samples were placed into numbered bags measuring 9×12 cm made of monofilament open nylon wire with a pore size of 40 microns. The sample bags were placed in a polyester mesh bag (45×45 cm with a 90 cm length of rope to anchor it to the cannula) weighed down with a plastic bottle (250 mL) filled with gravel to keep the samples in the liquid strata of the rumen. Bags were added according to the ‘gradual addition/all out’ schedule and were incubated for 0, 2, 4, 8, 12, 16, 24, and 48 h, respectively. The number of bags incubated for each sample was increased based on incubation time to ensure an adequate amount of residue remained for analysis. All treatments for every incubation period were implemented in two runs in five heifers. For each cow at each run, there were 2, 2, 2, 2, 2, 3, 5, and 8 bags for 0, 2, 4, 8, 12, 16, 24, and 48 h incubations, respectively. After incubation, the bags were rinsed in a bucket of tap water before washing to remove the debris and stop fermentation. Washed bags were then dried at 65°C in a forced air oven for 48 h. Dry samples were stored in a refrigerated room (4°C) until chemical analysis.

### Chemical analysis

Samples were analyzed according to the procedure of the AOAC for DM (method 930.15), ash (method 942.05), ether extract (EE, method 920.39), CP (method 984.13) (8400 kjeltec analyzer unit, Foss Tecator, Hoganas, Sweden) [13]. Acid detergent insoluble N (ADIN) (Licitra et al [14]), neutral detergent insoluble N (NDIN) (Licitra et al [14]) were determined, non-protein N (NPN) and soluble CP (SCP) was measured according to the method suggested by Roe et al [15]. The Starch was analyzed using Megazyme Total starch assay kit. Acid detergent insoluble protein (ADIP) and neutral detergent insoluble protein (NDIP) were calculated as: ADIP = 6.25×ADIN and NDIP = 6.25×NDIN. Acid detergent fiber, neutral detergent fiber (NDF), and acid detergent lignin were analyzed by Ankom filter bag method (ANKOM 2000 Filter Bag technique, Ankom Technology, Fairport, NY, USA) with a modified fat extraction procedure, which included an initial 2 h ether extraction along with the standard Ankom acetone fat extraction protocol, to prevent the high fat content of the flaxseed samples from giving erroneously high values for the fiber measurements [16]. The NDF was analyzed without the addition of sodium sulfite and with the inclusion of heat stable α-amylase as previously described [16]. AA profiles were analyzed by 6 N HCl and oxidized digestions via AA Autoanalyzer (Hitichi 8890, Tokyo, Japan).

### Fractionation of protein according to Cornell Net Carbohydrate and Protein System

The CP subfractions were divided into 3 parts based on CNCPS [17]. The characterizations of the CP fractions as applied in this system are as follows: directly available (soluble) protein (PA) is NPN, fraction PB is true protein, and fraction PC is unavailable protein. Fraction PB is further partitioned into three parts (PB1, PB2, and PB3) that are believed to have different rates of degradation in the rumen. Fraction PC is the ADIN, which is highly resistant to breakdown by microbial and enzymes, and it is assumed to be unavailable for the animal. The relative rumen degradation rates of the five protein fractions have been described as follows: fractions PA is assumed to be infinity, fraction PB1 is 1.20 to 4.00/h, fraction PB2 is 0.03 to 0.16/h, fraction PB3 is 0.0006 to 0.0055/h. Fraction PC is considered to be undegradable [17]. Estimated contents for truly digestible CP (tdCP) and total digestible nutrient maintenance level (TDN1x) were explored by using a summative approach (Weiss et al [18]) from NRC [10].

### Intestinal protein digestion determination

Intestinal digestibility of rumen undegraded feed protein (dRUP %) is also referred to as *in vitro* pepsin-pancreatin digestibility of rumen undegradable protein. Rumen incubation residues of CM, SBM, and RSM from different origins at 16 h incubation times were collected for* in vitro* pepsin-pancreatin digestion tests. Dried incubation residues as previously described were ground to pass through a Wiley mill (1 mm screen) and kept in airtight plastic bags prior to tests of CP digestion parameters using the *in vitro* pepsin-pancreatin digestion method (Calsamiglia and Stern [19]). Briefly, sample containing 15 mg of residual N was placed into a 50 mL incubation tube and incubated for 1 h in 10 mL of 0.005 mol/L HC1 solution (pH = 1.9) containing 5 g/L of pepsin (≥12,000 U/g, Sigma P-7012, Sigma Chemical, St. Louis, MO, USA). All tests were run in triplicates. The incubating media was then neutralized to pH of 7.8 with 0.1 M NaOH solution before 20 mL pancreatin solution (Sigma P-7545, Sigma Chemical, USA) was added, and incubated at 38°C for 24 h in a shaking water bath. During incubation, all tubes were vibrated thoroughly every 8 hours. After 24 h incubation, 3 mL of trichloroacetic acid (100%, wt/vol) solution was added to the tubes to stop enzymatic action and precipitate the undigested protein. At 15 minutes after adding trichloroacetic acid, the incubating tubes solution was added, and the samples were vibrated thoroughly once again. The incubating media tube was then centrifuged at 10,000×g for 15 min, the sediment was transferred completely to a drying vial, taken out and further dried at 65°C for 48 hours in a forced air oven to measure of N content (Leco FP-528, Joseph, MI, USA).

### Mid infrared microspectroscopy

The protein spectral features were studied at Tianjin Agricultural University molecular spectroscopy lab with Lambda FTIR-7600 (Adelaide, Australia) following standard lab procedures. Spectra were generated in transmission mode with the mid-IR (4,000 to 800 cm^−1^) portion of the electromagnetic spectrum by 64 co-added scans with a spectral resolution of 4 cm^−1^. Spectral analysis (protein secondary structures) was done with OMNIC 7.5 (Thermo-Nicolet, Madison, WI, USA) software. Spectra were generated from randomly selected regions of feed.

### Statistical analysis

#### Chemical profile and protein subfraction studies

Statistical analyses were performed using the PROC MIXED procedure of SAS (version 9.2) [20]. The model used for the analysis was: Yij = μ+Fi+eij, where, Yij was an observation of the dependent variable ij; μ was the population mean for the variable; Fi was the difference of the feed type, as a fixed effect, and eij was the random error associated with the observation ij. The comparison of SBM, CM, and RSM was carried out using Contrast statement in SAS.

#### Protein secondary structure study

Statistical analyses were performed using the MIXED procedure of SAS (version 9.2) [20]. The model used for the analysis was: Yij = μ+Ti+S(T)j+eij, where, Yij was an observation of the dependent variable ij; μ was the population mean for the variable; Ti was the difference of the feed type, as a fixed effect, S(T)j is the seeds nested within treatments, as a random effect, and eij was the random error associated with the observation ij.

#### Rumen degradation and intestinal digestibility studies

Statistical analyses were performed using the PROC MIXED procedure of SAS (version 9.2) [20]. The model used for the analysis was: Yijk = μ+Ti+Sk+eijk, where, Yijk was an observation of the dependent variable ijk; μ was the population mean for the variable; Ti was the difference of the feed type, as a fixed effect, Sk was the run effect, as a random effect, and eijk was the random error associated with the observation ijk.

For all statistical analyses, significance was declared at p < 0.05 and trends at p≤0.10. The Tukey-Kramer method was used in multi-comparison after variance analysis and a SAS macro called “pdmix800” (Saxton 1998) was used to denote the letter for each treatment mean at the significance level of 0.05. Normality of residual of each variable was tested by using PROC UNIVARIATE in SAS 9.2 with Normal and Plot options [21].

## RESULTS AND DISCUSSION

### Difference in basic chemical profile among soybean meal, canola meal, and rapeseed meal

The parameters of DM and ash were significantly different (p<0.05) among SBM, CM, and RSM, with significantly higher DM content and lower ash content in SBM and CM than that of RSM (Table 1). That means there was more organic matter in SBM and CM than RSM. The content of EE did not show a significant difference among the feed samples (p>0.05). The results from this study showed a similar trend in DM and Ash values for CM to those reported by Theodoridou and Yu [8]. Based on the findings of our research, SBM might contain the best chemical profile density, RSM the worse, with CM in between.

Significant differences were observed in CP among the three types of feedstuffs (p<0.05), CP content in SBM was higher than CM and RSM. The parameters associated with protein, such as NDIP, ADIP, and SCP, did not differ significantly (p> 0.05) among SBM, CM, and RSM.

### Detecting changes in amino acid

Results of the special compounds of protein (AA) are presented in Table 2. The content of all AA showed significant differences (p<0.05) among the SBM, CM, and RSM. Aspartic acid, threonine, serine, isoleucine, and lysine was highest in SBM, while their content in RSM was lowest (p<0.05). The content of glutamic, proline, glycine, alanine, valine, leucine, tyrosine, phenylalanine, histidine, arginine, and tryptophan in SBM was significantly higher than that of CM and RSM (p<0.05). SBM had lower (p<0.05) content of methionine and cystine than CM and RSM. For total AA, there was no significant difference between CM and RSM (p>0.05), but total AA content in SBM was significantly higher than that of CM and SBM (p<0.05). Our results were closely in agreement with those reported by previous study [22]. Balanced AA profiles of digestible RUP are quality indicators in the evaluation of protein feed ingredients in ruminant diets [23]. Histidine (His) may be the first-limiting AA if the diet is mainly made up of corn, SBM, cottonseed meal, corn silage and corn based distillers dried grains with solubles [24]. In addition, Piepenbrink and Schingoethe [25] reported that valine (Val) has become the first-limiting AA for CM. We found a similar change regulation between His and Val in the feed samples. In SBM, His and Val were significant greater than that of CM and RSM (p<0.05). No significant difference was found in His and Val comparing CM with RSM (p>0.05). This result suggested that SBM had better AA balance than CM and RSM. Based on the data from this work, it is concluded that SBM had a greater AA content and components than CM and RSM.

### Detecting changes in protein subfractions

The results of CP subfractions partitioned by CNCPS and digestible nutrients using NRC [10] model are presented in Table 3. The CP subfractions partitioned by CNCPS did not show differences (p>0.05) among SBM, CM, and RSM. Since each subfraction was highly related to protein utilization and availability in ruminants, this result implied that SBM, CM, and RSM did not differ significantly regarding protein utilization. The tdCP contents in SBM was significantly higher (p<0.05) than that of CM and SBM. Although significant differences were not achieved, the result of total digestible nutrient (TDN_1×_, at one times maintenance estimated for NRC dairy model 2001) still showed a tendency that TDN_1×_ content was SBM>CM>RSM (p = 0.06).

### Protein degradation and intestinal digestion

As shown in Table 4, feed type had no effect (p>0.05) on the effective degradation rate of DM in oil-seed-meals, nor the effective degradation rate of CP. A high NDIP proportion could reflect higher content of slowly degradable protein fraction in the rumen [26]. NDIP and ADIP was numerically higher in RSM but no significant differences were observed among the three feedstuffs used in this study. According to the values in Table 4, the effective crude protein degradability in RSM was the lowest compared with that in SBM and CM, which was in good agreement with the above-mentioned that the PC fraction was related to the protein degradability in the rumen. Therefore, the protein part of RSM was more difficult to be utilized in the rumen of cow than was the CM’s and SBM’s.

There was significant difference (p = 0.05) in intestinal di gestibility of protein among SBM, CM and RSM. RSM was shown to have lowest intestinal digestibility of rumen undegradable protein, and SBM had the highest with CM in between. Combined with the previous conclusion, tdCP contents in SBM were higher than that in CM, and SBM and CM were both greater than that in RSM. The results revealed that both rumen degradation and intestinal digestibility were different among SBM, CM, and RSM, and these phenomena may be caused by diverse protein sources.

### Detecting changes in protein molecular structure features

Results of protein secondary structure analysis are shown in Table 5. The protein internal structure α-helix and β-sheet were modeled and identified using secondary derivative function based on amide I component peaks centered at ca. 1,650 and 1,625 cm^−1^, respectively. We found the absorbance peak height and area intensities of protein amide I, amide II, α-helix and β-sheet height as well as their ratio in SBM, CM, and RSM. All parameters of protein secondary structure were significantly different (p<0.05) among SBM, CM, and RSM except amide I to amide II area ratio (p = 0.53). Amide I area, Amide I peak height, Amide II area, α-helix and β-sheet height in RSM were significantly lower than those in SBM and in CM (p<0.05). Reports showed that amide I region mainly resulted from C=O stretching vibration and C-N stretching vibration. The region of amide II was primarily associated with NH in-plane bending and C-N stretching, also related to C=O stretching, C-C stretching and N-C stretching [27]. For Amide II peak height, SBM was highest of the 3 types of feedstuffs (p<0.05), and RSM was lowest while CM fell in between. Amide I to Amide II peak height and α-helix to β-sheet height ratio in SBM were significantly lower than those in CM and RSM (p<0.05). Amide I and Amide II can be used to assess the protein conformation and protein molecular l structure [28]. Different α-helix, β-sheet height in the protein inherent structure might result in different protein nutritive value. Theodoridou and Yu [12] stated that a strongly negative relationship (p<0.05) existed between ratio of α-helix to β-sheet and tdCP content in CM and presscake. This conclusion agreed with our test results, namely, SBM had greater protein content which could be used by ruminants based on the good secondary protein structure compared with CM and RSM. Although SBM feedstock had a higher CP than CM and RSM, the results of protein secondary structure analysis demonstrated that SBM and CM had similar protein characteristics and they are better than RSM.

SBM is a common dietary material owing to its good pro tein content and stable quality. However, considering the feed cost and economic benefit, the use of CM and RSM as oil by-product alternatives to SBM has become commonplace. Many studies compared the effects of substituting SBM by CM on milk production and composition [29,30] and discovered that feeding these protein supplements may be as effective as feeding SBM to lactating dairy cows. Our experiment revealed that SBM and CM had similar internal structure of protein, and their protein quality and molecular structure were better than RSM (p<0.05). Therefore, this study suggests that both SBM and CM can provide similar benefits as protein source for dairy cattle, while the protein quality and utilization of RSM was worse than SBM and CM in ruminants.

## CONCLUSION

The results of this study indicated that different types and sources of feedstocks showed differences in protein nutrient and availability in ruminants. The differences of SBM, CM, and RSM in protein nutrient and utilization profiles may be accounted for by distinction of protein internal structural construction. Using the Fourier transform infrared spectroscopy analytical technique, the changes of protein structure in feedstuffs with different protein source were revealed and identified. Our research suggested that RSM and CM had similar protein quality in the view of molecular structure, and their protein internal structural construction was better than that of SBM.

## Figures and Tables

**Table 1:** Chemical profiles of soybean meal, canola meal and rapeseed meal

Items	SBM	CM	RSM	SEM	p value	Contrast p value		

						SBM vs CM	SBM vs RSM	RSM vs CM
Basic chemical profiles								
DM (g/kg)	932.9	927.7	914.3	2.910	0.01	0.28	<0.01	0.03
Ash (g/kg DM)	67.1	72.3	85.7	4.12	0.01	0.28	<0.01	0.04
EE (g/kg DM)	15.1	21.8	12.5	3.02	0.20	0.19	0.53	0.09
Protein profile								
Total CP (g/kg DM)	503.8	398.0	425.0	15.54	0.002	<0.01	<0.01	0.15
NDIP1) (g/kg DM)	73.1	51.3	147.8	41.81	0.31	0.74	0.23	0.18
ADIP2) (g/kg DM)	62.4	28.0	88.4	33.56	0.52	0.51	0.58	0.27
SCP (g/kg DM)	314.6	245.3	279.0	45.5	0.61	0.34	0.58	0.63
NPN (g/kg DM)	130.2	141.9	123.2	16.80	0.76	0.66	0.76	0.48
NDIP (g/kg CP)	146.8	129.3	343.8	131.80	0.26	0.90	0.17	0.18
ADIP (g/kg CP)	125.6	85.2	101.3	16.01	0.46	0.31	0.46	0.63
SCP (g/kg CP)	627.2	609.2	657.2	95.57	0.94	0.90	0.82	0.75
NPN (g/kg CP)	258.3	354.9	291.4	38.87	0.32	0.15	0.54	0.32
NPN (g/kg SCP)	446.7	604.9	440.9	91.54	0.46	0.29	0.96	0.28

SBM, soybean meal; CM, canola meal; RSM, rapeseed meal; SEM, standard error of the mean; DM, dry matter; EE, ether extract; CP, crude protein; NDIP, neutral detergent insoluble protein; ADIP, acid detergent insoluble protein; SCP, soluble crude protein; NPN, non-protein nitrogen.

1) NDIP = 6.25×NDIN.

2) ADIP = 6.25×ANIN.

**Table 2:** Amino acid contents in soybean meal, canola meal, and rapeseed meal

Item (g/kg DM)	SBM	CM	RSM	SEM	p value	Contrast p value		

						SBM vs CM	SBM vs RSM	RSM vs CM
Aspartic acid	51.6	25.8	24.4	0.40	<0.01	<0.01	<0.01	0.02
Threonine	18.9	16.7	15.9	0.15	<0.01	<0.01	<0.01	0.02
Serine	23.3	15.9	15.2	0.18	<0.01	<0.01	<0.01	0.04
Glutamic	79.8	61.1	61.4	0.62	<0.01	<0.01	<0.01	0.78
Proline	23.5	22.4	22.5	0.26	0.05	0.03	0.03	0.71
Glycine	19.7	18.7	18.2	0.21	0.01	0.02	<0.01	0.23
Alanine	20.5	16.8	16.7	0.16	<0.01	<0.01	<0.01	0.74
Cystine	6.1	9.1	8.3	0.40	0.01	<0.01	0.01	0.24
Valine	20.7	18.3	18.0	0.01	<0.01	<0.01	<0.01	0.55
Methionine	6.0	7.6	7.7	0.12	<0.01	<0.01	<0.01	0.59
Isoleucine	19.4	13.7	13.1	0.12	<0.01	<0.01	<0.01	0.02
Leucine	35.4	26.1	25.4	0.25	<0.01	<0.01	<0.01	0.15
Tyrosine	18.3	11.7	10.8	0.37	<0.01	<0.01	<0.01	0.15
Phenylalanine	18.9	11.3	10.9	0.65	<0.01	<0.01	<0.01	0.69
Histidine	10.1	8.5	8.4	0.16	<0.01	<0.01	<0.01	0.84
Lysine	27.4	22.9	16.3	2.47	0.05	0.26	0.02	0.13
Arginine	31.4	21.0	17.6	1.31	<0.01	<0.01	<0.01	0.13
Tryptophan	5.7	4.6	4.2	0.21	<0.01	<0.01	<0.01	0.27
Total AA1)	436.8	331.6	314.8	4.57	<0.01	<0.01	<0.01	0.053

DM, dry matter; SBM, soybean meal; CM, canola meal; RSM, rapeseed meal; SEM, standard error of mean, the same as below.

1) Total AA, sum of aspartic, threonine, serine, glutamic, proline, glycine, alanine, cystine, valine, methionine, isoleucine, leucine, tyrosine, phenylalanine, histidine, lysine, arginine, and tryptophan.

**Table 3:** Protein subfractions profiles using Cornell Net carbohydrate and protein system and digestible nutrient of dry matter in soybean meal, canola meal and rapeseed meal

Items	SBM	CM	RSM	SEM	p value	Contrast p value		

						SBM vs CM	SBM vs RSM	RSM vs CM
Fractions of protein by CNCPS1)								
PA (% CP)	25.83	35.49	29.14	3.887	0.32	0.15	0.54	0.32
PB1 (% CP)	36.90	25.43	36.58	9.302	0.67	0.44	0.98	0.45
PB2 (% CP)	22.60	26.16	6.88	10.970	0.46	0.83	0.32	0.28
PB3 (% CP)	3.42	5.82	13.90	3.413	0.14	0.65	0.07	0.17
PC (% CP)	12.41	7.11	20.48	7.608	0.51	0.65	0.45	0.28
Component digestible nutrient and digestible nutrient at maintenance level2) (% DM)								
tdNFC	15.64	23.86	17.56	3.313	0.31	0.15	0.68	0.25
tdCP	47.88	38.68	38.97	1.429	0.01	<0.01	0.01	0.89
tTDN_1×_	76.75	73.94	62.51	3.438	0.06	0.60	0.03	0.07

SBM, soybean meal; CM, canola meal; RSM, rapeseed meal; SEM, standard error of mean; CNCPS, Cornell Net Carbohydrate and Protein System; CP, crude protein; DM, dry matter; NDF, neutral detergent fiber; ADF, acid detergent fiber.

1) Protein subfractions using CNCPS include: PA, fraction of CP that is instantaneously solubilized at time zero; PB1, fraction of CP that is soluble in borate-phosphate buffer and precipitated with trichloroacetic acid; PB2, calculated as total CP minus the sum of fractions PA, PB1, PB3 and PC; PB3, calculated as the difference between the portions of total CP recovered with NDF and ADF; PC, fraction of CP recovered with ADF and is considered to be undegradable. It contained proteins associated with lignin and tannins and heat-damaged proteins such as the Mailard reaction products.

2) tdNFC, truly digestible non-fiber carbohydrate; tdCP, truly digestible crude protein; TDN_1×_, total digestible nutrient at one times maintenance estimated for NRC dairy model 2001.

**Table 4:** *In situ* degradation kinetics profile of dry matter and crude protein and intestinal digestibility of rumen-undegradable protein in soybean meal, canola meal and rapeseed meal

Items	SBM	CM	RSM	SEM	p value	Contrast p value		

						SBM vs CM	SBM vs RSM	RSM vs CM
*In situ* rumen degradation kinetics of DM								
a (%)1)	1.09	−0.38	−0.18	1.651	0.79	0.57	0.58	0.94
b (%)2)	87.69	60.10	46.28	9.941	0.06	0.12	0.02	0.39
c (h^−1^)3)	0.03	0.07	0.05	0.006	0.01	<0.01	0.03	0.04
DMED	51.90	46.45	32.87	6.059	0.14	0.57	0.06	0.19
*In situ* rumen degradation kinetics of CP								
a (%)	−2.51	0.89	1.37	2.591	0.52	0.41	0.31	0.91
b (%)	538.28	67.84	132.64	89.542	0.02	0.02	0.02	0.64
c (h^−1^)	0.001	0.05	0.02	0.008	0.02	0.01	0.22	0.04
CPED	57.90	49.65	43.00	5.659	0.23	0.37	0.10	0.46
Intestinal digestibility of RUP								
dRUP (%)	61.01	52.53	50.10	2.554	0.05	0.07	0.02	0.54

SBM, soybean meal; CM, canola meal; RSM, rapeseed meal; SEM, standard error of mean; DM, dry matter; DMED, effective dry matter degradability (%); CP, crude protein; CPED, effective crude protein degradability (%); RUP, rumen-undegradable protein; dRUP, digestibility of rumen undegradable protein.

1) a, rapidly degraded fraction.

2) b, potentially degraded fraction.

3) c, fractional degradation rate of b.

**Table 5:** Secondary structure analysis of protein in soybean meal, canola meal and rapeseed meal

Items	SBM	CM	RSM	SEM	p value	Contrast p value		

						SBM vs CM	SBM vs RSM	RSM vs CM
Amide I area	5.06	4.51	3.16	0.432	0.01	0.27	<0.01	0.03
Amide I height	0.06	0.05	0.04	0.003	0.01	0.21	<0.01	0.03
Amide II area	2.04	1.74	1.23	0.095	<0.01	0.08	<0.01	0.01
Amide II height	0.03	0.03	0.02	0.002	<0.01	0.02	<0.01	0.02
A I+A II1)	6.61	6.66	4.62	0.482	0.04	0.94	0.02	0.03
A I/A II area1)	2.48	2.61	2.58	0.083	0.53	0.32	0.40	0.78
A I/A II height1)	1.70	1.92	2.00	0.027	<0.01	<0.01	<0.01	0.05
α-helix height	0.05	0.05	0.04	0.003	0.02	0.40	0.01	0.03
β-sheet height	0.06	0.05	0.05	0.003	0.01	0.08	<0.01	0.03
ratio:α/β	0.94	1.03	1.01	0.018	0.03	0.02	0.03	0.47

SBM, soybean meal; CM, canola meal; RSM, rapeseed meal; SEM, standard error of mean.

1) A I+A II, Amide I area+Amide II area; A I/A II area, ratio of Amide I area to Amide II area; A I/A II height, ratio of Amide I height to Amide II height.
